# Creating an automated trigger for sepsis clinical decision support at emergency department triage using machine learning

**DOI:** 10.1371/journal.pone.0174708

**Published:** 2017-04-06

**Authors:** Steven Horng, David A. Sontag, Yoni Halpern, Yacine Jernite, Nathan I. Shapiro, Larry A. Nathanson

**Affiliations:** 1 Department of Emergency Medicine, Beth Israel Deaconess Medical Center, Harvard Medical School, Boston, Massachusetts, United States of America; 2 Department of Electrical Engineering and Computer Science, Institute for Medical Engineering and Science, Massachusetts Institute of Technology, Cambridge, Massachusetts, United States of America; 3 Google, Cambridge, Massachusetts, United States of America; 4 Department of Computer Science, Courant Institute of Mathematical Sciences, New York University, New York, New York, United States of America; Garvan Institute of Medical Research, AUSTRALIA

## Abstract

**Objective:**

To demonstrate the incremental benefit of using free text data in addition to vital sign and demographic data to identify patients with suspected infection in the emergency department.

**Methods:**

This was a retrospective, observational cohort study performed at a tertiary academic teaching hospital. All consecutive ED patient visits between 12/17/08 and 2/17/13 were included. No patients were excluded. The primary outcome measure was infection diagnosed in the emergency department defined as a patient having an infection related ED ICD-9-CM discharge diagnosis. Patients were randomly allocated to train (64%), validate (20%), and test (16%) data sets. After preprocessing the free text using bigram and negation detection, we built four models to predict infection, incrementally adding vital signs, chief complaint, and free text nursing assessment. We used two different methods to represent free text: a bag of words model and a topic model. We then used a support vector machine to build the prediction model. We calculated the area under the receiver operating characteristic curve to compare the discriminatory power of each model.

**Results:**

A total of 230,936 patient visits were included in the study. Approximately 14% of patients had the primary outcome of diagnosed infection. The area under the ROC curve (AUC) for the vitals model, which used only vital signs and demographic data, was 0.67 for the training data set, 0.67 for the validation data set, and 0.67 (95% CI 0.65–0.69) for the test data set. The AUC for the chief complaint model which also included demographic and vital sign data was 0.84 for the training data set, 0.83 for the validation data set, and 0.83 (95% CI 0.81–0.84) for the test data set. The best performing methods made use of all of the free text. In particular, the AUC for the bag-of-words model was 0.89 for training data set, 0.86 for the validation data set, and 0.86 (95% CI 0.85–0.87) for the test data set. The AUC for the topic model was 0.86 for the training data set, 0.86 for the validation data set, and 0.85 (95% CI 0.84–0.86) for the test data set.

**Conclusion:**

Compared to previous work that only used structured data such as vital signs and demographic information, utilizing free text drastically improves the discriminatory ability (increase in AUC from 0.67 to 0.86) of identifying infection.

## Introduction

### Background

Clinical informatics interventions in the form of alerts, reminders, and clinical decision support systems have effectively changed clinician behaviors across a broad spectrum of diseases [1,2,3]. The emergency department (ED) is an obvious setting to deploy these technologies given the high information burden and large numbers of critically ill patients that require time-dependent interventions [4]. Unfortunately, clinical informatics interventions are difficult to tailor and implement in the emergency department [5]. Vital signs and patient demographics are commonly used, but are often neither sensitive nor specific. Instead, decision support systems often rely on structured data (also known as coded data) to trigger these systems, data that is difficult to collect and therefore rarely collected as part of routine clinical care. Overburdened emergency departments most in need of these systems are unlikely to allocate additional resources to enter additional coded data. In contrast, free text data is already routinely collected and contains a rich source of information about a patient, but is almost never used to drive clinical informatics interventions to support clinical care [6].

### Importance

Sepsis, a severe form of infection, is responsible for significant morbidity, mortality, and costs to patients in our healthcare system, leading to an estimated 751,000 deaths nationally [7]. Early protocolized care for sepsis can improve outcomes, but emergency departments are still struggling to consistently implement early protocolized care for sepsis [8,9]. Clinical decision support systems have been shown to improve compliance to these treatment protocols in ICUs by guiding physicians through predefined workflows [10]. However, unlike the ICU and other inpatient settings, ED patients do not have documented diagnoses and other structured data that can be used to trigger these pathways. In order for these sepsis clinical decision support systems to be effective in the ED, we need a reliable way to trigger this system for patients at risk for sepsis. This triggering method must be done early and in real-time before critical decisions and treatments are initiated.

Since nursing triage is the first point of contact for patients in the ED, it is the ideal time to implement early sepsis triggers. A naïve method to trigger these pathways would be to ask the triage nurse whether a patient is eligible for a protocol. In fact, some electronic medical records collect this type of structured data at triage and are able to inherently trigger these types of systems. Although manual data entry of structured data is easy to implement, it is not a scalable methodology. Asking a triage nurse whether this patient qualifies for a sepsis protocol is okay. Asking them to also answer additional questions for ten, one hundred, and eventually one thousand protocols is not sustainable. We instead propose a novel application of machine learning to use routinely collected data at triage such as patient demographics, vital signs, free text chief complaint, and free text nursing assessment (also called the triage note) to trigger a protocol. Such a method would not impose any additional workload or change the workflow for the triage nurse.

### Goals of this investigation

We present a novel clinical application of machine learning methods to trigger clinical decision support at ED triage. We specifically focus on identifying patients with ED ICD-9-CM defined infection for the purpose of triggering sepsis clinical decision support. However, these methods are easily generalizable to any type of decision support using data available at ED triage. Whereas previous work on triggering clinical decision support at ED triage made use only of carefully curated structured data such as vital signs [11,12,13], our primary goal is to demonstrate that it is feasible and beneficial to also use routinely collected free text data at triage to predict infection. We employ several state-of-the-art machine learning methods together with the free text, demographics, and vital signs. We do not aim to do an exhaustive comparison of machine learning methods, but rather to demonstrate the significant improvement in predictive performance that is possible when using routinely collected unstructured data such as clinical notes.

## Materials and methods

### Overview

We conducted a retrospective, observational cohort study of consecutive patients at a 55,000 visits/year Emergency Department over a 50 month period to derive a machine learning algorithm to identify infection at triage. We collected ED triage text, triage vital signs, and ED ICD-9-CM codes from the electronic medical record and trained machine learning algorithms to predict ICD-9-CM defined infection using incrementally larger subsets of features. The study was approved by the Beth Israel Deaconess Medical Center Institutional Review Board (protocol number 2011P-000356). A waiver of informed consent was granted as it met the requirements described in 45 CFR 46.116(d) of the US Department of Health and Human Services Policy for Protection of Human Research Subjects.

### Setting and selection of participants

The study was performed in a 55,000 visits/year Level I trauma center and tertiary academic teaching hospital. All consecutive ED patient visits between 12/17/2008 and 2/17/2013 were included in the study. No visits were excluded.

### Outcome measures

The primary outcome measure was diagnosed infection in the emergency department. A patient was defined to have an infection if one of their ED ICD-9-CM discharge diagnoses contained an ICD-9-CM diagnosis defined by the Angus Sepsis ICD-9-CM abstraction criteria [7]. The Angus Sepsis ICD-9-CM abstraction criteria is a list of ICD-9-CM codes often used in sepsis research that correspond to diagnoses consistent with infection.

### Data collection and processing

We collected 12 features from data available at ED triage, shown in Table 1, as well as the ED ICD-9-CM discharge diagnoses from the electronic medical record. Each feature was modeled as listed in the data type. Acuity was modeled as a 5-level ordinal variable rather than a continuous variable to account for non-linearity. We refer to the first 10 features in the table as vital sign data for brevity, even though it includes some demographic data.

**Table 1:** Data types used in models and amount of missing or out of range data.

Feature	Data Type	Missing or Out of Range Data
Age	Continuous	0%
Gender	Binary	0%
Acuity	5-level ordinal	2.7%
Systolic blood pressure	Continuous	4.9%
Diastolic blood pressure	Continuous	5.2%
Heart rate	Continuous	4.6%
Pain scale	Continuous	6.6%
Respiratory rate	Continuous	6.5%
Oxygen saturation	Continuous	5.6%
Temperature	Continuous	6.7%
Free text chief complaint	Text	0%
Free text nursing assessment	Text	2.9%

To perform tokenization on chief complaints and nursing assessments, we first separate punctuation from the beginnings and ends of words and then consider a token to be any sequence of symbols, separated by white space (e.g., “s/p” and “h/a” are both considered words). We then applied bigram detection. For example, the common bigram “chest pain” is hyphenated (“chest-pain”) so that it is considered a single word. Negation detection is then used to append “_neg” after negated terms. For example, “no chest-pain” and “no fever, chills” would be substituted with “chest-pain_neg” and “fever_neg, _neg chills_neg”. We used a custom negation detection algorithm that is based on NegEx [14], but has additional negation termination words that are optimized for ED triage nursing assessments. See S1 Appendix for implementation details of the tokenization, bigram, and negation detection algorithms.

Data validation was automatically performed on all covariates to ensure all variables were correctly formatted and in range. Vital signs that were missing or out of predefined physiological ranges were automatically imputed with a physiologically normal value. After imputation, all values were normalized to lie in the range [0,1]. The presence of missing values was relatively rare and also presented in Table 1. When alphanumerical data appeared where numerical data was expected, only the first valid digits were used. For example, “101.9 rectally” was changed to “101.9”. No rows were excluded from analysis. No indicator variable was used to denote an imputed value. Further details of the data imputation are provided in the S1 Appendix.

One example of an out of range normal that was imputed is a patient with a pain score of 1000. The visual analog pain scale is intended to be a value between 0 and 10. However, patients at times report pain scores much greater than 10. A pain score larger than 10, such as 11 or 1000 does not mean a patient has more pain than a patient with a pain score of 10. There are many reasons for patients to report pain scores higher than the maximum value. Some patients falsely believe that if they do not exaggerate their pain, they may not receive pain medications. Other patients believe over-reporting their pain will offer secondary advantages such as more rapid care. Some patients, though, simply are in severe pain and use extreme pain scores to communicate the severity of their pain.

Another example of an out of range value would be a typographic error. For example, a heart rate of 811 was found for one patient. Since a heart rate above 400 is physiologically impossible, a heart rate of 811 can never exist. More than likely, this was a typographic error. Since the value is outside of the range that we predefined to be physiologically realistic for heart rate, we automatically impute this to a physiologically normal value of 80.

### Model building

Patients were randomly allocated to fixed train (n = 147,799; 64%), validate (n = 46,187; 20%), and test (n = 36,950; 16%) data sets. Our primary models were constructed by machine learning using a linear support vector machine (SVM) that optimizes the area under the ROC curve (AUC). We used the open-source SVM^*perf*^ software package [15]. The data set has substantial class imbalance, since infection only occurs in 14% of the patients. This learning algorithm automatically controls for class imbalance by directly optimizing a lower bound on the AUC, rather than focusing on classification accuracy [15]. For comparison purposes, we additionally learned models using L2-regularized logistic regression, naïve Bayes, and random forests, using the open-source Scikit-Learn software [16]. For all learning algorithms, model derivation was first performed on the train data set. The validate data set was used to optimize over model parameters. The test data set, a holdout sample, was then used to test the internal generalizability of the model with the highest AUC on the validate data set. When we report train and validate results, we also report them for the model with the highest AUC on the validate data set.

We trained four models (see Table 2). The first model, *vitals*, has a feature vector derived solely from the 10 vital signs and demographic covariates. All subsequent models utilize free text in addition to the vitals. In the second model, *chief complaints*, we used the chief complaint along with vitals. In the third model, *bag of words*, we used both the chief complaint and the nursing assessment along with vitals. For both the second and third models, we included one feature for each word in the vocabulary whose value is the term frequency, defined as the number of occurrences of that word in a patient’s free text. The vocabulary consists of all words that appear at least 5 times in the entire dataset. For the bag of words model, the vocabulary consists of 15,240 words.

**Table 2:** Features used for the predictive models.

	Vital Signs	Patient Demographics	Chief Complaint	Nursing Assessment
Vitals Model	X	X		
Chief Complaint Model	X	X	X	
Bag of Words Model	X	X	X	X
Topic Model	X	X	X	X

**Fig 1:**
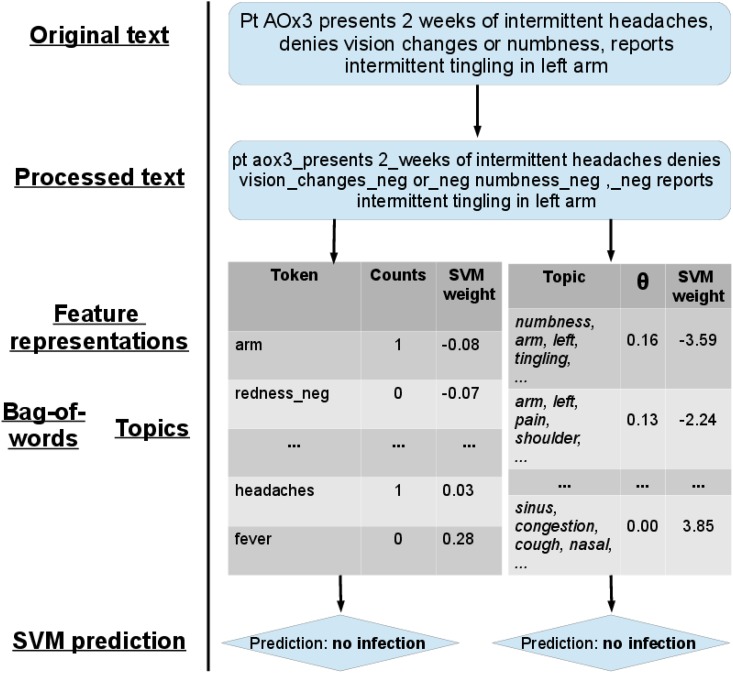
Pipeline for natural language processing and prediction. Our algorithm first takes as input a triage note and processes it by applying tokenization followed by bigram and negation detection, the latter using a customized version of the NegEx tool [14]. The processed text is then transformed into a set of features. The Bag-of-Words features count how many times each word in our vocabulary appears in the processed note, and the Topic model features (derived using the Mallet [17] tool) measure how much certain topics are represented in the note. A Support Vector Machine (SVM) is then trained on these sets of features to determine whether the patient presents an infection, using the SVM^*perf*^ software [15].

In the fourth model, *topics*, clinical free texts were processed by learning a set of 500 “topics” that frequently occur across ED patients. Then, given a patient’s chief complaint and triage text, we inferred a distribution of topics for each patient, providing a low-dimensional representation of each patient’s free text. In addition to vitals, we had one feature for each topic, whose value is the probability of a patient having that topic. We used the open-source MALLET software to learn the topic model [17]. The topic proportions of each document were concatenated with demographic information and vital signs to form the final feature vector used in classification. Topics with values of less than 0.001 were set to 0. An illustration of the overall pipeline is given in Fig 1. Further implementation details are provided in S1 Appendix.

### Primary data analysis

Means with 95% confidence intervals were reported for age, temperature, heart rate, systolic blood pressure, and diastolic blood pressure. Medians with interquartile ranges were reported for severity, respiratory rate, oxygen saturation, pain scale, admission days, and ICU days. Significance testing was performed using T-tests for parametric data, Wilcoxon rank sum for non-parametric data, and Fisher’s Exact test for proportions.

The area under the ROC curve (AUC) was calculated for each of the four models to measure discriminatory power. We also report positive predictive value (PPV), sensitivity, and specificity at the optimal cutoff point that balances the tradeoff between sensitivity and specificity. This optimal cutoff point is defined as the threshold which maximizes Youden’s J statistic (Sensitivity + Specificity—1). To better understand the models’ calibration, we plot for each model and for each predicted probability range 0 to 0.1, 0.1 to 0.2, and so on, the fraction of patients with this predicted probability of infection that truly had an infection. We obtain a predicted probability from the SVM models by performing logistic regression using a bias term and a single feature corresponding to the continuous-valued prediction, a technique known as Platt scaling [18].

Statistical analysis was performed using JMP (JMP, Version 8. SAS Institute Inc., Cary, NC, 1989–2009) and SAS (SAS, Version 9.1.3. SAS Institute Inc., Cary, NC, 2002–2003). The SAS %roc macro version 1.7 was used to calculate AUC, 95% confidence intervals, and p-values.

## Results

### Characteristics of study subjects

A total of 230,936 patient visits were included in the study. Patients with infection (n = 32,103; 14%) were slightly older, had a higher temperature, faster heart rate, higher respiratory rate, lower systolic blood pressure, lower diastolic pressure, and were more frequently admitted, more frequently admitted to the ICU, and more likely to die within 28 days. These patient characteristics are reported in Table 3. The majority of triage notes have between 15 and 30 tokens.

**Table 3:** Statistics of data set.

	No Infection (*n = 198*,*833; 86%*)	Infection (*n = 32*,*103; 14%*)
Age—mean yrs. (95% CI)	49.7 (49.6,49.7)	54.5 (54.3,54.7)
Male gender—no. (%)	91,158 (46%)	13,546 (42%)
Severity—median ESI [IQR]	3 [2–3]	3 [2–3]
Temperature—mean Degrees Fahrenheit (95% CI)	97.7 (97.7,97.7)	98.1 (98.1,98.1)
Heart Rate—mean Beats per minute (95% CI)	84.3 (84.2,84.3)	88.4 (88.2, 88.6)
Respiratory Rate—median bpm [IQR]	17 [16–18]	18 [16–18]
Oxygen Saturation—median [IQR]	100 [98–100]	99 [97–100]
Systolic Blood Pressure—mean mm Hg (95% CI)	134.8 (134.7,134.9)	132.4 (132.1,132.6)
Diastolic Blood Pressure—mean mm Hg (95% CI)	77.6 (77.6,77.7)	74.6 (74.4,74.7)
Pain Scale—median 10 point Likert Scale [IQR]	4 [0–8]	5 [0–8]
Admitted—no. (%)	59,590 (30.0%)	16,339 (50.9%)
ICU admission—no. (%)	11,488 (19.3%)	3,250 (19.9%)
ICU admission—median no. days [IQR]	2 [1–4]	2 [1–4]
28 day in-hospital mortality—no. (%)	2,470 (1.2%)	725 (2.3%)

### Model performance

**Fig 2:**
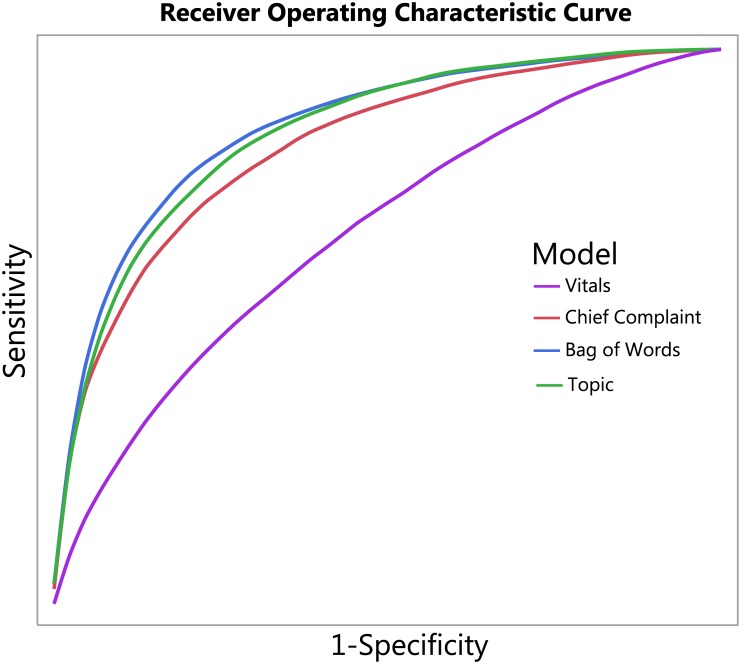
Receiver operating characteristic curve. Vitals—Age, Gender, Severity, Temperature, Heart Rate, Respiratory Rate, Oxygen Saturation, Systolic Blood Pressure, Diastolic Blood Pressure, Pain Scale. Chief Complaint—Chief Complaint + Vitals. Bag of Words—Vitals + Chief Complaint + Triage Assessment. Topics—Vitals + Chief Complaint + Triage Assessment

**Fig 3:**
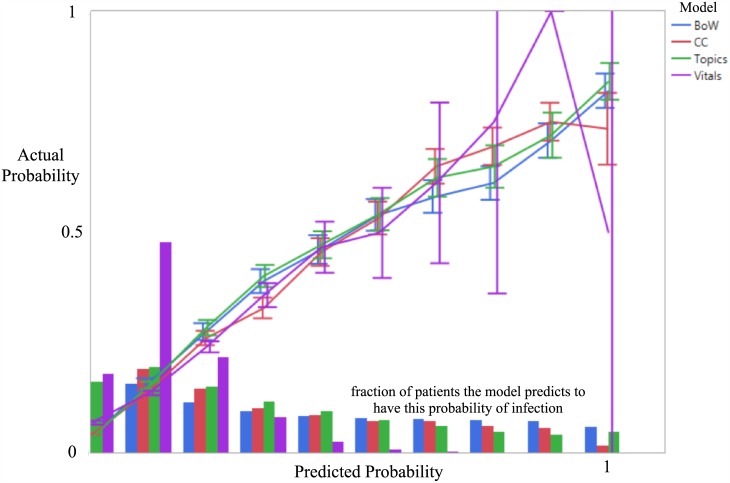
Calibration plots. We assess the models’ calibration by plotting for each predicted probability range, in increments of 0.1, the fraction of patients with this predicted probability of infection that truly had an infection. Perfect calibration would correspond to the straight line from (0,0) to (1,1). We additionally show bar plots of the number of predictions made by each method within each probability interval. The Vitals model, which has the least data to go on, makes very few predictions of infection with probability greater than 0.5, leading to very large confidence intervals toward the upper right of the plot. The Bag of Words and Topics models are better calibrated, and are particularly accurate for the highest risk patients. Vitals—Age, Gender, Severity, Temperature, Heart Rate, Respiratory Rate, Oxygen Saturation, Systolic Blood Pressure, Diastolic Blood Pressure, Pain Scale. CC—Chief Complaint + Vitals. BoW (Bag of Words)—Vitals + Chief Complaint + Triage Assessment. Topics—Vitals + Chief Complaint + Triage Assessment

The area under the ROC curve (AUC) for the vitals model, which used only vital signs and demographic data, was 0.67 for the training data set, 0.67 for the validation data set, and 0.67 (95% CI 0.65–0.69) for the test data set. The AUC for the chief complaint model which also included demographic and vital sign data was 0.84 for the training data set, 0.83 for the validation data set, and 0.83 (95% CI 0.81–0.84) for the test data set. The best performing methods made use of all of the free text. In particular, the AUC for the bag-of-words model was 0.89 for training data set, 0.86 for the validation data set, and 0.86 (95% CI 0.85–0.87) for the test data set. The AUC for the topic model was 0.86 for the training data set, 0.86 for the validation data set, and 0.85 (95% CI 0.84–0.86) for the test data set. These results are presented in Table 4, and the full receiver-operator curves are given in Fig 2. The calibration plots are given in Fig 3. All models achieve excellent calibration. The confidence intervals for the model based on demographic and vital sign data are particularly large toward the larger probability ranges because the model predicts a probability of infection larger than 0.5 only for very few patients. The topic model and the bag-of-words model, on the other hand, are able to make use of the full range of probabilities.

**Table 4:** Performance characteristics for the SVM models.

	Train (*n = 147*,*799; 64%*)				Test (*n = 36*,*950; 16%*)			
	AUC	PPV	Sensitivity	Specificity	AUC	PPV	Sensitivity	Specificity
Vitals	0.67	0.22	0.56	0.67	0.67	0.22	0.56	0.68
CC	0.84	0.34	0.78	0.75	0.83	0.32	0.75	0.75
BoW	0.89	0.40	0.83	0.80	0.86	0.38	0.78	0.79
Topics	0.86	0.34	0.81	0.75	0.85	0.34	0.80	0.75

Vitals—Age, Gender, Severity, Temperature, Heart Rate, Respiratory Rate, Oxygen Saturation, Systolic Blood Pressure, Diastolic Blood Pressure, Pain Scale CC—Chief Complaint + Vitals BoW—Bag of Words model using Vitals + Chief Complaint + Triage Assessment Topics—Topic Model using Vitals + Chief Complaint + Triage Assessment * All Test AUCs have a 95% CI of +-0.02. A validation data set (n = 46,187; 20%) was also used as an intermediary data set between train and test to select regularization parameters. We do not show these here, for brevity.

We show in Table 5 a comparison of the SVM with several alternative machine learning algorithms. Logistic regression, when the data points are reweighted to account for class imbalance, performs similarly to the SVM on all feature sets. Random forests obtain an AUC of 0.70 on the vital signs and demographic data, improving on the linear models (SVM and L2-regularized logistic regression), both of which obtained an AUC of 0.67. However, once the text data is included, the linear models perform similarly to random forests. Naïve Bayes consistently underperformed the other machine learning methods across the three settings considered.

**Table 5:** Comparison with alternative machine learning algorithms.

	Linear SVM	Logistic Regression	Naïve Bayes	Random Forests
Vitals	0.67	0.67	0.65	0.70
BoW	0.86	0.86	0.83	0.87
Topics	0.85	0.84	0.70	0.83

Comparison of area under the Receiver-Operator Curve (AUC) on Test (n = 36,950) for various machine learning algorithms. Parameters such as regularization constants (linear SVM, logistic regression) and minimum number of samples per leaf (random forests) were chosen by evaluating the AUC on the validate data set. All results have a 95% CI of +-0.02.

Vitals—Age, Gender, Severity, Temperature, Heart Rate, Respiratory Rate, Oxygen Saturation, Systolic Blood Pressure, Diastolic Blood Pressure, Pain Scale

BoW—Bag of Words model using Vitals + Chief Complaint + Triage Assessment

Topics—Topic Model using Vitals + Chief Complaint + Triage Assessment

We next did an error analysis to understand how the SVM models performed on specific patient cohorts. Table 6 shows the sensitivity, i.e. the fraction of patients with infection that are predicted to have infection, for each cohort. Admission can be considered a surrogate for severity (more so for admission to the ICU), and thus these patients are more likely to have had severe sepsis. The topic model has the best sensitivity, 0.81, for predicting infection at triage time for patients that will later be admitted to the ICU. All models are significantly worse at predicting urinary tract infection than pneumonia.

**Table 6:** Sensitivity of test data set by patient subset.

	Not Admitted (*n = 24*,*795; 67%*)	Admitted to ICU or Floor (*n = 12*,*155; 33%*)	Admitted to ICU (*n = 2*,*317; 6%)*	Admitted to Floor (*n = 9*,*838; 27%*)	Pneumonia (*n = 969; 3%*)	Urinary Tract Infection (n = *1*,*112; 3%*)
CC	0.74	0.76	0.79	0.74	0.81	0.66
BoW	0.78	0.78	0.76	0.78	0.79	0.68
Topics	0.79	0.8	0.81	0.8	0.83	0.72

All models use the ‘Vitals’ features—Age, Gender, Severity, Temperature, Heart Rate, Respiratory Rate, Oxygen Saturation, Systolic Blood Pressure, Diastolic Blood Pressure, Pain Scale

CC—Chief Complaint + Vitals

BoW—Bag of Words model using Vitals + Chief Complaint + Triage Assessment

Topics—Topic Model using Vitals + Chief Complaint + Triage Assessment

### Analysis of free text models

Since we used machine learning to learn a linear model, we can analyze the beta coefficients or weights to better understand which words are being used by the different models to predict infection. In Table 7 we show for the chief complaint model several of the most positive (i.e., when the word is present in the chief complaint, patient *more* is likely to have an infection) and negative (i.e., patient *less* likely to have an infection) words. Table 8 shows the most positive and negative words for the bag of words model, which uses both the chief complaint and the triage assessment.

**Table 7:** SVM model learned using chief complaints.

Weight	Word
2.18	cellulitis
2.10	sore_throat
2.09	st *(sore throat)*
2.05	abcess (misspelling of abscess)
2.00	uti *(urinary tract infection)*
1.98	abscess
1.85	dysuria
1.61	pneumonia
1.59	cyst
1.58	infection
-1.10	migraine
-1.11	etoh *(ethanol*, *for drunkenness)*
-1.16	injury
-1.26	laceration
-1.26	status_post_assault
-1.30	epistaxis
-1.34	lac *(laceration)*
-1.42	status_post_mvc
-1.48	mvc *(motor vehicle crash)*

Most positive (indicative of infection) and negative (suggesting no infection) words from the model built by machine learning using only chief complaints. In parentheses/italics are our annotations, to point out what these acronyms are typically used for.

**Table 8:** SVM model learned using bag-of-words.

Weight	Word
0.98	cellulitis
0.80	uti
0.79	redness_swelling
0.78	sore_throat
0.77	abscess
0.73	diverticulitis
0.72	abscess
0.70	dysuria
0.66	st
0.65	erythema
0.20	swelling
-0.29	swelling_neg
-0.35	pancreatic
-0.36	eye
-0.36	bleed
-0.37	etoh
-0.37	epistaxis
-0.38	pancreatitis
-0.39	injury
-0.57	mvc

Most positive (indicative of infection) and negative (suggesting no infection) words used by the model built by machine learning using the bag-of-words model on triage notes.

In Table 9 we show the 11 topics with the most positive weights (most predictive of a patient having an infection), and the 11 topics with the most negative weights (most predictive of a patient *not* having an infection). These 22 topics are a subset of the 500 topics that are automatically discovered by unsupervised learning of the topic model. Each topic is described by the words that are most frequently seen in a patient’s chief complaint or triage assessment for patients with that topic.

**Table 9:** SVM model learned using topics.

Weight	Topic (described by most frequent words)
**11.00**	redness, cellulitis, left, leg, swelling, area, rle, arm, lle, increased, erythema
**8.38**	abcess, buttock, area, drainage, axilla, groin, painful, thigh, left, hx, abcesses, red, boil
**8.15**	cellulitis, abx, pt, iv, infection, po, keflex, antibiotics, leg, treated, started, yesterday
**7.13**	red, swollen, touch, warm, painful, area, left, infection, swelling, tender, slightly, hot
**6.65**	abscess, left, area, fevers_neg, axilla, cyst, size, i&d, lesion, lump, swelling, mass, thigh
**6.60**	pna, pneumonia, cxr, wbc, dec_num, transfer, rll, anon_1140, rehab, fever, lll, recent
**6.40**	sore_throat, throat, st, voice, secretions, swallowing, pain, swallow, difficulty_swallowing
**5.90**	uti, pt, cipro, abx, dx, started, treated, recent, bactrim, fever, c/o, recently, infection
**5.69**	pna, cough, sob, pneumonia, cxr, recent, dx, abx, fever, r/o, fevers, bronchitis, recently, tb
**5.64**	dysuria, hematuria, uti, c/o, urination, pain_neg, burning, denies, frequency, urgency,
**2.12**	wound, check, eval, pt, abcess, wick, i&d, abscess, drained, removal, returns, fevers_neg
**-1.80**	pain, ankle, weight, bearing, left, foot, swelling, knee, wt, injury, bear, unable_bear
**-3.44**	struck, bike, car, ped, accident, bicycle, loc_neg, pain, riding, hit, bicyclist, pt, fell, c/o
**-3.59**	numbness, arm, left, tingling, facial, hand, leg, weakness, side, sided, c/o, today, resolved
**-3.63**	epistaxis, bleeding, nose, pt, bleed, pressure, bleeding_neg, blood, on_coumadin, stopped
**-3.64**	status_post_mvc, mvc, car, restrained_driver, loc_neg, passenger, neck, driver, front, side
**-3.89**	fall, status_post_fall, fell, ladder, feet, pain, landed, ft, 10, loc_neg, back, approx, foot, steps
**-3.90**	gi, bleed, status_post, colonoscopy, endoscopy, procedure, today, esophageal, upper, scope
**-4.26**	playing, injury, ball, soccer, pt, game, football, hit, hockey, player, struck, baseball, loc_neg
**-4.29**	mvc, trauma, gsw, basic, mcc, 21, status_post_mvc, transfer, rollover, rm, room, stabbing
**-4.91**	etoh, found, vomiting, apparently, drunk, drinking, denies, friends, trauma_neg, triage,
**-5.18**	watching, tv, sitting, sudden_onset, movie, television, smoked, couch, pt, pot, 5pm, theater

Most positive (indicative of infection) and negative (suggesting no infection) topics from the model built by machine learning using features derived from the topic model on triage notes.

## Discussion

Patients in the emergency department often have time-dependent disease processes where delays in diagnosis or treatment can lead to poor outcomes. Clinical decision support targeted at emergency department workflows must therefore also be timely. Conventional methods to trigger decision support such as the administration of a medication, ordering of a test, or assignment of diagnosis code are already too late since these are exactly the targets for decision support in the emergency department.

Automating decision support using routinely collected data remains a “Grand Challenge” of clinical decision support [19]. Several previous authors have considered the use of machine learning for identifying [20] or better managing sepsis [21]. However, whereas our paper focuses on the *very* early detection of sepsis, at Emergency Department triage, previous work considered identification of sepsis much later in a patient’s hospital stay, using data such as laboratory test results or continuous vital signs that are not available in our setting [22,23,24,25]. Moreover, none of these previous works considered the use of free text data. Vital sign abnormalities are often used to trigger decision support for sepsis and other diseases [11,12,13] at triage time, but we show in this paper that they are neither sensitive nor specific. Using all available data, including free text, presents an opportunity to improve the performance of these decision support triggers [6,19,26].

Our research sought to determine the incremental benefit of utilizing free text in addition to vital signs to trigger sepsis clinical decision support. Even utilizing the small amount of free text found in chief complaints resulted in an improvement in AUC of the linear models from 0.67 (95% CI 0.65–0.69) to 0.83 (95% CI 0.81–0.84). Adding even more free text, using either a Bag of Words model or a Topic model continued to increase the AUC to 0.86 (95% CI 0.85–0.87) and 0.85 (95% CI 0.84–0.86), respectively. Specifically, we found that the free text in triage notes is particularly valuable for obtaining a broader context of the reason for the patient’s ED visit. In some cases this can help rule out the possibility of the patient having sepsis, such as in the following triage note:

“cantonese speaking with numness right arm blurred vision dizziness lack of focus SOB since8 am. tongue midline. no facial droop. same sxs as strok in 08.”

The symptoms described in this triage note suggest that the patient is likely suffering from a stroke, not a severe infection. In other cases, the text can provide evidence toward the patient having an infection, such as in the following triage note,

“89 yo f s/p esophageal hernia repair w/? g-tube placement now w/ c/o's n&v. family reports pt's appetite is decreased, no BM x3d. generally not feeling well, had a bad day.”

which is consistent with a patient having a surgical-site infection. Looking at the vital signs alone would give significant less information in cases like these.

The linear SVM models shown in Tables 7 and 8 make sense clinically: the most positive weighted words include “cellulitis”, “sore throat”, and “abscess”, all words indicative of an infection, and the most negative words include “laceration”, “etoh” (ethanol, for drunkenness), and “mvc” (motor vehicle crash), other reasons for why a patient may come to an emergency department. Moreover, the learning algorithm’s ability to automatically discover the predictive utility of synonyms and misspellings of words, such as “abcess” (misspelling of “abscess”) and “st” (abbreviation of “sore throat”) demonstrate the advantage and simplicity of using machine learning with clinical big data. Many of the discovered topics shown in Table 9 correspond to well-known reasons for why a patient may come to an emergency department, such as bike accidents, sports injuries, drunkenness, cellulitis, and sore throat. We see that the support vector machine is able to distinguish infection topics from non-infection topics.

We had expected that using a machine learning algorithm that modeled non-linear interactions between the features might improve our methods ability to predict infection. Indeed, using random forests, an ensemble of decision trees, improves AUC from 0.67 to 0.70 when only considering the continuous-valued demographics and vital signs. However, random forests did not improve prediction accuracy once the free text from the chief complaints and triage note were added to the feature set, even when used together with the topic model which would seem to be well suited for such an approach. Given the simplicity, interpretability, and competitive performance of the linear models (either from the SVM or the L2-regularized logistic regression), they appear to be the best suited method for this setting.

There was very little drop in AUC between evaluations performed over the test and training data set for all of the models (0 for the vitals model, 0.01 for the chief complaint model, 0.03 for the bag of words model, and 0.01 for the topic model). This suggests very little overfitting and good internal generalizability to new data. Using a bag of words model for free text results in over 15k features. Thus, it was important to use regularization within the support vector machine to minimize the overfitting effect of such a large feature vector. To better understand the relative strengths of the different models, we performed an error analysis (sensitivity analysis) among different subgroups in our study population. We specifically looked at patients that were discharged, admitted to the floor, admitted to the ICU, had a diagnosis of pneumonia, and had a diagnosis of an urinary tract infection (UTI). The chief complaint model had the highest degree of variability, ranging in sensitivity from 0.66–0.81 (stdev 0.05). The bag of words model had a smaller range of sensitivity, from 0.68–0.79 (stdev 0.04). The topic model had the smallest variability in sensitivity, from 0.72–0.83 (stdev 0.03). All models had poor sensitivity for predicting UTI. Future work should consider the incorporation of additional features such as laboratory test results, once they become available, which could improve predictive performance for UTI and other subtler conditions.

There are a number of limitations to this study. First, we used the ED ICD-9-CM discharge diagnoses as our outcome measure, which may have misclassified patients. We attempted to limit bias by using a standardized abstraction criterion commonly used in sepsis research [7]. However, patients may have been suspected of having an infection in the ED and ultimately may have had an alternative diagnosis, or the diagnosis of infection may not have been abstracted properly. Although an outcome measure based on formal chart review would have been methodically more rigorous, it was not feasible for a study of this size (>200,000 patient visits). These considerations withstanding, we submit that our standardized approach will yield valid results. Secondly, we have not performed any normalization of the free text data to correct for misspellings or synonyms. Although previous work with chief complaints used normalization, we specifically chose not to do this in order to show that such preprocessing is not necessary when dealing with big data. Rather than manually creating rules and dictionaries for normalization, which can be a time consuming process that would potentially need to be repeated for different applications or settings, we instead use machine learning to learn the predictive value of each of the individual misspellings and synonyms. We believe it is a particular strength of our study that we can obtain reasonable results without creating manual rules or dictionaries. Lastly, while we internally validated our results, external validation is warranted. It will be interesting to discover whether the same model may be applied to another institution without any modification, or whether reliable prediction first requires training on local free text.

In conclusion, accurate triggering of clinical decision support will become increasingly more important as clinical decision support becomes more integrated into electronic medical records. Since decision support has the potential to interrupt the clinical workflow, every attempt should be made to ensure that all eligible patients receive the decision support (sensitivity), and that non-eligible patients are not mistakenly targeted (specificity) leading to alert fatigue. Our study shows that utilizing free text in addition to vital sign and demographic information alone will drastically improve the discriminatory ability (increase in AUC from 0.67 to 0.86) of these triggers to identify infection, improving both sensitivity and specificity. In the coming years, commercial electronic medical record vendors will begin to allow the importing of predictive models for triggering clinical decision support. Our work emphasizes the need for the vendors to support features derived from clinical text.

## Supporting information

S1 Appendix Detailed description of methods used for natural language processing and machine learning. (DOC)
