# Supplementary material for: Creating an automated trigger for sepsis clinical decision support at emergency department triage using machine learning
Source: PLoS One. 2017 Apr 6;12(4):e0174708. doi: 10.1371/journal.pone.0174708 (PMC5383046; doi:10.1371/journal.pone.0174708)
Supplement: S1 Appendix — (DOC) [file pone.0174708.s001.doc]

S1 Appendix

In this section we provide further implementation details of the natural language processing and machine learning.

**Tokenization:**

To tokenize the text into individual words or tokens, we apply the following operations :

1. Notes are transformed into lower case,
2. *“p.o.”* is transformed into *“p_o”* to avoid dealing with punctuation issues,
3. Any number with a decimal part, such as *“20.14”* is transformed into the token “*dec_num*”,
4. Punctuations in the list *“. , ; + - ( )”* are surrounded by white spaces.

Finally, any sequence of characters between two white spaces is considered to be a token. We applied tokenization and bigram detection to the triage assessment and chief complaints fields of the notes, and then applied negation detection to the triage assessment. We wrote the software for the tokenization and bigram detection using Python, and used a modification of the NegEx software for negation detection.

**Bigram detection:**

A bigram is a pair of adjacent words that typically refer to a phrase. We aggregate the most useful bigrams, such as *“chest pain”*, *“mental status”* or *“kidney stones”*, into single tokens. We also obtain several common tri-grams by having the bigram detection algorithm ignore extremely common words and punctuation, such as *“of”* (*“shortness of breath”* becomes *“shortness_breath”*), *“-”* (*“c - spine”* becomes *“c_spine”*), or *“and”* (*“calm and cooperative”* becomes *“calm_cooperative”*).

We use a two-step method to rate a bigram's significance. First, we count how often any sequence of two words is present in the training data set, and only selected those that arose more than 500 times as candidate significant bigrams. Then, we score all candidate bigrams *“w1 w2”* according to the measure :

$$f\left(w1,w2\right)=\frac{counts\left(w1 w2\right)}{counts\left(w1\right)*counts\left(w2\right)}$$

where *counts(u v)* denotes how many times word *u* was followed by word *v*, and *counts(w)* is the number of occurrences of word *w* in the training data set. The advantage of this measure is that it avoids selecting bigrams that are common because both of their words are common (but have no particular significance appearing next to each other), such as *“now reports”* or *“also complains”*. The importance of the filter requiring that potential bigrams appear at least 500 times is to prevent two adjacent words from scoring highly simply because they only occur a handful of times in the data (e.g., if “cat dog” appeared only once in the text, f(“cat”, “dog”) would be 1, which is the maximum possible value of f). We then select the 120 highest-scoring candidate bigrams, and replace any occurrences of them in the data by the aggregated tokens. For example, the note

*“pt states took antibiotic x 5 days after initiation of* ***sore throat*** *and sx resolved”*

is transformed into

*“pt states took antibiotic x 5 days after initiation of sore_throat and sx resolved”.*

The threshold of 120 was chosen after looking at the sorted list and noticing that beyond the 120^th^, the vast majority of the phrases were not clinically meaningful bigrams, but were rather phrases such as “presents complaining” and “o male” (121^st^ and 122^nd^).

**Negation Detection:**

We used a simple variant of the NegEx algorithm for negation detection. We consider that words are negated if they are situated between a negation word, such as ***no*** (see below for full list), and a termination token***,*** such as a period (see below for full list). Contrary to NegEx, we do not automatically stop the scope of a negation word after a given number of tokens. We found that this is more appropriate for the concise notes that are typical of triage assessments.

For example, the note

*“Foot is red swollen warm to the touch,* ***denies*** *fever at home* ***.*** *Hx multiple infections”*

is transformed into

*“Foot is red swollen warm to the touch, denies fever_neg at_neg home_neg .* *Hx multiple infections”.*

In this example,*“denies”* is a negation word and *“.”* is a termination token, hence the phrase *“fever at home”* is negated.

*The negation words and termination tokens that we use are listed in the following table:*

| **Negation words** | **Termination tokens** |
| --- | --- |
| denies  no  non  not  without  unable | .  -  ;  +  and  aox3  but  complains  did  except  has  per  pt  reports  secondary  states |

To evaluate our modifications to NegEx, we manually labeled the words in 200 triage note sentences from the validate data set with their negation status. Using the modifications described above, the precision increased from 0.699 (NegEx) to 0.833 (modified NegEx) and recall improved from 0.875 (NegEx) to 0.982 (modified NegEx). The overall F1 measure improved from 0.777 (NegEx) to 0.901 (modified NegEx). We expect that these modifications to NegEx will be more broadly useful for other applications of natural language processing on emergency department triage nursing notes.

We found that the performance characteristics on the test data were not statistically significantly different after performing bigram and negation detection, compared to using the unprocessed free text data. However, performing bigram and negation detection significantly improves the interpretability of the resulting models.

**Removing Rare Tokens and Punctuation:**

After applying tokenization, bigram detection and negation to the corpus of notes, every token that does not appear at least five times in the corpus was removed, along with punctuation. Negated punctuation was not removed. This filter was used only for computational reasons. When learning using the SVM, the regularization (which is used to prevent overfitting) leads to the weights of the very infrequent words being set to values close to zero. As a result, the performance characteristics are the same as if we had simply omitted these infrequent words from the beginning. As a result, it is standard practice in statistical natural language processing, and machine learning more broadly, to remove very rare features prior to training.

**Unsupervised Topic Models:**

Unsupervised topic models were trained using Mallet to learn an LDA (Latent Dirichlet Allocation) model with 20, 100, 500, and 1000 topics on the preprocessed textual corpus of triage notes and chief complaints. Only patients from the training set were used in this training step. Early in our investigation we found that beyond 500 topics the AUC on the validate data set did not substantially improve and interpretability got worse, so we chose to use 500 topics for the remainder of the analyses. Unless otherwise specified, all parameters of Mallet were set to default settings. In addition to removing the rare tokens as described above, common tokens that do not convey much information on their own are also removed. The default list of common words from Mallet was used, augmented to include the negations of each word (eg. “the” and “the_neg”). Once the topic model was learned, it was used to infer the topic proportions of each document. The number of iterations of Gibbs sampling for this inference step was set to 1000. The topic proportions of the document were concatenated with demographic information and vital signs to form the final feature vector used in classification. Topics with values of less than 0.001 were set to 0.

The procedure of training topic models in Mallet uses a random initialization. Ten different random initializations were used and the topic model that provided features that predicted with the highest AUC on the validate data set was used.

**Supervised Learning of Classifiers**

Classifiers to maximize the area under the curve (AUC) measure were trained using SVM*^perf^* . Learning a support vector machine classifier requires the user to choose a cost parameter, C. Values of C were tested increasing by factors of 10 from 10^-3^ to 10^6^ and the classifier with the highest AUC on the validate data set was used.

The comparisons to logistic regression, naïve Bayes, and random forests were performed using the open-source Scikit-Learn software, version 0.16.1, in Python . As with the SVM, the validate data set was used to test generalization and to tune parameters. For logistic regression, we tested values of the regularization constant C increasing by factors of 10 from .01 to 100. For both Vitals and Topics we used a Gaussian naïve Bayes model. Better results for BoW were obtained using a Bernoulli naïve Bayes model, omitting the continuous-valued features (Scikit-Learn does not provide an option for fitting naïve Bayes models with a combination of discrete and continuous features). For random forests, each predictor used an ensemble of 200 trees (estimators), and we reweighted the classes to account for the imbalanced data set. For each data set, we tested different settings of the number of samples per leaf, a means of preventing the classifiers from overfitting. We obtained the best results using the ‘entropy’ (information gain) criteria to split trees and by learning deep trees, choosing among 1, 2, 10, 100, and 200 samples per leaf. The optimal choices on the validate data set were 100 samples per leaf (Vitals), 10 samples per leaf (Topics), and 2 samples per leaf (BoW). Using 2000 trees substantially increased training time and did not improve results.

**Details of Data Imputation**

Vital signs that were missing or out of predefined physiological ranges were automatically imputed with a physiologically normal value. Below we give, for each vital sign, the lower and upper bounds of the ranges that we considered, and the imputed values that we used if the reported value was out of range:

|  | Lower bound | Upper bound | Imputed |
| --- | --- | --- | --- |
| Heart rate | 30 | 220 | 80 |
| Systolic blood pressure | 40 | 280 | 120 |
| Diastolic blood pressure | 20 | 150 | 80 |
| Respiratory rate | 5 | 60 | 12 |
| Temperature | 80 | 106 | 98.6 |
| Oxygen Saturation | 68 | 100 | 97 if <68 100 if >100 |
| Pain | 0 | 10 | 5 if <0  10 if >10 |

These choices for imputation were made based on clinical judgement, together with an analysis of the distribution of the observed data for each of the vital signs.

1. Manning, C.D. and H. Schutze, *Foundations of statistical natural language processing*. 1999, Cambridge, Mass.: MIT Press. xxxvii, 680 p.

2. Chapman, W.W., et al., *A simple algorithm for identifying negated findings and diseases in discharge summaries.* Journal of biomedical informatics, 2001. **34**(5): p. 301-310.

3. McCallum, A., *MALLET: A Machine Learning for Language Toolkit*. 2002.

4. Joachims, T., *A Support Vector Method for Multivariate Performance Measures*, in *ICML*. 2005, ACM Press. p. 377-384.

5. Fabian Pedregosa, G.V., Alexandre Gramfort, Vincent Michel, Bertrand Thirion, Olivier Grisel, Mathieu Blondel, Peter Prettenhofer, Ron Weiss, Vincent Dubourg, Jake Vanderplas, Alexandre Passos, David Cournapeau, Matthieu Brucher, Matthieu Perrot, and Édouard Duchesnay, *Scikit-learn: Machine Learning in Python.* Journal of Machine Learning Research, 2011. **12**: p. 2825-2830.
